# Heparin to prevent recurrent placenta-mediated pregnancy complications in women with antiphospholipid syndrome: a systematic review

**DOI:** 10.1016/j.rpth.2026.106634

**Published:** 2026-05-08

**Authors:** Valérie L.B.I. Jansen, Hanke M.G. Wiegers, Dorien M. Salet, Thijs E. van Mens, Saskia Middeldorp

**Affiliations:** 1 Department of Vascular Medicine, Amsterdam Cardiovascular Sciences, Amsterdam Reproduction and Development, Amsterdam University Medical Centers, University of Amsterdam, Netherlands; 2 Department of Internal Medicine, Radboud University Medical Center, Nijmegen, Netherlands; 3 Department of Medicine—Thrombosis and Haemostasis, Leiden University Medical Center, Leiden, Netherlands

**Keywords:** antiphospholipid syndrome, HELLP syndrome, heparin, low-molecular-weight heparin, pre-eclampsia

## Abstract

**Background:**

For women with antiphospholipid syndrome (APS) and placenta-mediated complications, the efficacy of heparin during subsequent pregnancies is uncertain.

**Objectives:**

This study systematically reviewed high-quality evidence on efficacy of low-molecular-weight heparin (LMWH) to prevent recurrent placenta-mediated pregnancy complications in women with APS.

**Methods:**

We searched Embase, MEDLINE, and CENTRAL to identify randomized controlled trials (RCTs) that assessed use of LMWH compared with any control in pregnant women with APS and previous placenta-mediated pregnancy complications (intrauterine growth restriction; birth of a small for gestational infant; preeclampsia; hemolysis, elevated liver enzymes, and low platelets syndrome; late fetal loss; and placental abruption). The primary outcome was recurrence of placenta-mediated pregnancy complications.

**Results:**

We identified 4007 records and included 2 RCTs. The TIPPS trial randomized eligible women to dalteparin or no dalteparin; 21 women with APS were included in this systematic review. The FRUIT trial randomized 32 women with APS to dalteparin and aspirin or aspirin only. The primary outcome of this systematic review occurred in 4 of 16 women in the intervention group of the FRUIT trial and in 3 to 6 of 16 women in the control group (risk difference, 6.25%; 95% CI, −22.3% to 34.8%; ranging to −12.5%; 95% CI, −44.3% to 19.3%, due to absent reporting on coinciding complications). The trials did not report on the outcome and population as defined in the current review in a manner that allowed pooling the primary outcome.

**Conclusion:**

There is no high-quality evidence from RCTs that demonstrates a beneficial effect of LMWH in women with APS and placenta-mediated pregnancy complications without a history of recurrent miscarriage.

## Essentials

• Obstetric antiphospholipid syndrome (APS) manifests with miscarriages or placenta-mediated complications.
• Guidance on low-molecular-weight heparin (LMWH) to prevent placenta-mediated pregnancy complications in APS is inconclusive.
• Data are limited to a total of 53 randomized women and do not support LMWH to prevent recurrence.
• Evidence on LMWH for secondary prevention of placenta-mediated pregnancy complication in APS is insufficient.

## 1 Introduction

Antiphospholipid syndrome (APS) is a highly heterogeneous autoimmune disorder that is characterized by thrombosis and/or pregnancy morbidity, mediated by antiphospholipid antibodies (APLAs) [1]. There is a distinction between the thrombotic and obstetric phenotype. Within the obstetric phenotype, APS-defining pregnancy morbidity includes 3 separate clinical manifestations: unexplained recurrent miscarriage before the 10th week of gestation, unexplained fetal death at or beyond the 10th week, and premature birth before the 34th week due to severe preeclampsia or placental insufficiency. APS was defined in the revised Sapporo classification criteria by the combination of these clinical events, with persistent presence of APLA on 2 separate occasions [1]. Recently the Sapporo criteria have been updated in the 2023 American College of Rheumatology (ACR)/European Alliance of Associations for Rheumatology (EULAR) APS classification criteria, with the aim to have criteria with a high specificity to provide a strong foundation for future APS research [2]. These criteria consist of a weighted system that assigns a higher weight to preeclampsia and placental insufficiency compared with recurrent miscarriage or fetal loss. The ACR/EULAR criteria define single fetal loss ≥16 weeks as a clinical manifestation only, as opposed to ≥10 weeks in the revised Sapporo criteria. To classify recurrent miscarriage or fetal loss as APS, additional clinical manifestations are required. Indeed, the strength of the association of APLA with recurrent miscarriage is lower than with later, placenta-mediated complications such as preeclampsia, placental insufficiency leading to premature birth, and late fetal death [3]. Moreover, the underlying pathophysiology of early pregnancy loss and late placenta-mediated complications likely involves different processes [[4], [5], [6], [7]].

Women with obstetric APS are generally treated with antithrombotics, that is, a combination of low-dose aspirin and prophylactic-dose low-molecular-weight heparin (LMWH). In this study, LMWH refers to heparin, which may include LMWH or unfractionated heparin. Treatment with aspirin and LMWH is based on recommendations from guidelines that used aggregated evidence from low-quality randomized controlled trials (RCTs), suggesting that treatment with LMWH increases live birth rate in patients with APS and recurrent miscarriage [[8], [9], [10], [11], [12], [13]]. Whether this is also the case for women with APS and late placenta-mediated complications is uncertain. Considering the potentially distinct underlying pathophysiology and stronger APLA associations with placenta-mediated pregnancy complications compared with recurrent miscarriage, whether treatment effects observed in patients with APS and recurrent miscarriage extend to patients with APS and placenta-mediated pregnancy complications warrants evaluation. This systematic review aims to assess the efficacy of LMWH for preventing placenta-mediated pregnancy complications in women with APS and previous placenta-mediated pregnancy complications without a history of recurrent miscarriage.

## 2 Methods

This review was performed in accordance with the PRISMA guidelines (Supplementary Table 1), and the study protocol was registered in PROSPERO (ID CRD42022368709). The eligible population for this review consisted of pregnant women with APS, defined as persistent APLA according to original study definition, and a history of placenta-mediated pregnancy complications (intrauterine growth restriction or birth of a small for gestational age (SGA) infant; preeclampsia; eclampsia; hemolysis, elevated liver enzymes, and low platelets (HELLP) syndrome; late fetal loss; and placental abruption as defined by original study protocol). Women with APS and 2 or more miscarriages without placenta-mediated complications were not eligible since this research question was assessed in a previous systematic Cochrane review [13].

The intervention and comparator of interest were treatment with any type of heparin and a control arm without heparin (standard of care, aspirin, placebo, or no treatment). The primary outcome for this review was recurrence of placenta-mediated pregnancy complications, comprising intrauterine growth restriction or birth of an SGA infant, preeclampsia, eclampsia, HELLP syndrome, placental abruption, and late fetal loss. Late fetal loss was added to the outcome after publication of the protocol to best cover the evidence gap and was defined as fetal loss after 10 weeks of gestation. If the number of losses after 10 weeks of gestation were not available for extraction, the original study definition was adopted. Secondary outcomes are early pregnancy loss, preterm birth, and maternal adverse events, that is, venous thromboembolism (VTE) during pregnancy or puerperium, bleeding, and heparin-induced thrombocytopenia.

### 2.1 Search strategy

We systematically searched Embase, MEDLINE and The Cochrane Central Register of Controlled Trials up to May 31, 2025. The MEDLINE search included the Cochrane filter on RCTs. We did not apply restrictions on publication date. The complete search string is depicted in Supplementary Table 2. The search terms focused on APS, APLA, or thrombophilia; pregnancy or pregnancy complications; and heparin or LMWH. References of included studies were hand-searched for additional potentially relevant studies.

### 2.2 Selection of studies

Two authors (H.M.G.W. and V.L.B.I.J.) independently screened titles and abstracts of identified studies. Full texts from potentially eligible studies were screened in detail on inclusion criteria. We selected randomized or quasirandomized trials that included the eligible population. Studies with a mixed population of patients with APS and recurrent miscarriage and patients with APS and previous placenta-mediated pregnancy complications from which data on the latter group could not be separately extracted were excluded.

### 2.3 Data extraction

Two authors (H.M.G.W. and V.L.B.I.J.) independently extracted data from included studies with a standardized data extraction form. Extracted data included type LMWH, type of intervention in the control arm, number of patients with APS included, clinical and laboratory criteria for APS diagnosis, gravidity and obstetric history, mean age, pregnancy outcomes including placenta-mediated pregnancy complications as defined in the primary outcome, preterm birth and early pregnancy loss, bleeding, thrombotic complications, and heparin-induced thrombocytopenia.

### 2.4 Risk of bias assessment

The Cochrane RoB 2.0 was used to assess the risk of bias according to the Cochrane Handbook for systematic reviews of interventions [14]. Studies were qualified as low risk of bias, some concern, or high risk of bias. Two authors (D.M.S. and H.M.G.W.) independently evaluated the included studies. Discrepancies were resolved with help of a third author (V.L.B.I.J.).

### 2.5 Statistical analyses

We calculated risk differences (RDs) with 95% CIs. If the number of studies included would allow for meaningful pooling of data, a meta-analysis was planned using Knapp–Hartung random-effects model and potential publication bias assessment with a funnel plot.

## 3 Results

### 3.1 Selection of eligible studies

Our search strategy identified 4007 records, leaving 3689 records after removal of duplicates. Based on title and abstract screening 3683 records were excluded. Four reports were excluded after full-text screening based on study population (no APS, *n* = 3 [[15], [16], [17]] or recurrent miscarriage, *n* = 1 [18]) and study design (observational study [17]). The flowchart of the selection of studies is shown in Supplementary Figure 1.

### 3.2 Characteristics of included studies

We included 2 international multicenter RCTs: the TIPPS trial [19] and the FRUIT trial [20]. Study characteristics are listed in Table 1. The TIPPS trial included women with thrombophilia of which the relevant subgroup was included in this review.

**Table 1:** Characteristics of included studies.

Study	Type of study	Intervention	Control	Eligible gestational age for inclusion	Discontinuation of LMWH^a^	Total No. of women included	Women with APS included in this review	Laboratory criteria APS	Primary outcome
TIPPS [19]	Multicenter open-label RCT	Dalteparin 5000 IU once daily until 20 wk 5000 IU twice daily after 20 wk (+ aspirin at discretion of treating physician)	No antepartum dalteparin (+ aspirin at discretion of treating physician)	4-20 wk	After at least 37 wk gestational age	292	21	ACA IgG/IgM, >30 GPL/MPL U/mL; a-β_2_GP1 IgG/IgM, >20 GPL/MPL U/mL; positive LAC; 2 positive tests, interval undefined	Composite of VTE, severe or early-onset pre-eclampsia, SGA infant, and pregnancy loss
FRUIT [20]	Multicenter open-label RCT	Dalteparin 5000 IU once daily, adjusted for body weight+ aspirin 80 mg once daily	Aspirin 80 mg once daily	6-12 wk	Onset of labor or prior to cesarean section	32	32	ACA IgG/IgM > 10 GPL/MPL U/mL, positive LAC 2 positive tests; and interval of ≥6 wk	Hypertensive disorder of pregnancy onset <34 wk Hypertensive disorder of pregnancy irrespective of gestational age^b^

RCT, randomized controlled trial; LMWH, low-molecular-weight heparin; APLA, antiphospholipid antibody; ACA, anticardiolipin antibody; a-β_2_GP, anti–β_2_-glycoprotein 1 antibodies; LAC, lupus anticoagulant; VTE, venous thromboembolism.

a In both studies, all subjects (including those randomized to the control group) were prescribed LMWH until 6 weeks postpartum.

b Not prespecified in the protocol.

#### 3.2.1 Study population

The TIPPS trial included women with thrombophilia and at increased risk of VTE or with previous placenta-mediated pregnancy complications. Patients had to meet 1 or more criteria for high risk for VTE or for pregnancy complications. Obstetric risk factors included a history of ≥3 early pregnancy losses (<10 weeks), ≥2 pregnancy losses (>10 weeks), and ≥1 pregnancy losses (>16 weeks) or a history of preeclampsia irrespective of gestational age, birth of an SGA infant (birthweight <10th percentile), or severe placental abruption (leading to fetal distress, maternal shock, or maternal coagulopathy). VTE risk inclusion criteria comprised a history of provoked proximal VTE or calf vein thrombosis or superficial phlebitis or a family history of VTE in a first-degree family member. Women requiring anticoagulation, including women with APS and recurrent pregnancy loss were excluded. One of the women with APS was included based on a provoked proximal VTE and was excluded from this review. The VTE-related risk factor criteria comprised either risk factors that do not prompt APLA testing (such as superficial phlebitis, calf vein thrombosis, or a family history of VTE) or a history of provoked VTE. In the context of APLA positivity, a history of VTE warrants LMWH treatment and consequently would have led to exclusion. Thus, we assume that all other women with APLAs included in the TIPPS trial had prior placenta-mediated pregnancy complications (preeclampsia, birth of an SGA infant, placental abruption, or late pregnancy loss). Positive APLA was defined as 2 positive tests of 1 or more of the following: anticardiolipin IgG/IgM of >30 U/mL, anti-β_2_ glycoprotein IgG/IgM of >20 U/mL, or positive lupus anticoagulant. The time window between 2 positive tests was not defined.

The FRUIT trial included women with APS and a previous delivery before 34 weeks of gestation with preeclampsia (pregnancy-induced hypertension in combination with proteinuria >20 weeks of gestation), eclampsia (generalized nonepileptic convulsions), HELLP syndrome (lactate dehydrogenase ≥ 600 IU/L, alanine aminotransferase ≥ 70 IU/L, and platelet count <100 × 10^9^/L) or birth of an SGA infant (birthweight <10th percentile) [20]. Persistent APLA were defined as anticardiolipin of >10 GPL/MPL and/or positive lupus anticoagulant on 2 occasions at least 6 weeks apart.

#### 3.2.2 Intervention and control arm

The TIPPS trial was designed with a placebo control arm but switched to open label after 26 months due to slow recruitment. Participants were randomized before 20 weeks of gestation between dalteparin 5000 IU once daily and no dalteparin. The dose of dalteparin was increased to 5000 IU twice daily after 20 of weeks of gestation. Dalteparin was continued until at least 37 weeks of gestation. Concurrent use of aspirin was decided by the treating physician.

The design of the FRUIT trial was open label. Participants were randomized before 12 weeks of gestation between dalteparin 5000 IU once daily and low-dose aspirin or low-dose aspirin alone. The dose of dalteparin was adjusted for body weight (<50 kg, 2500 IU; >80 kg, 7500 IU). Dalteparin was stopped at onset of labor or prior to cesarean section and aspirin was stopped at 36 weeks of gestation. In both studies, all women, including those randomized to the control group, were treated with postpartum LMWH prophylaxis until 6 weeks postpartum.

### 3.3 Risk of bias

Risk of bias was assessed using The Cochrane RoB 2.0 tool. The risk of bias assessment is shown in Supplementary Figure 2. Both studies were classified as having some concerns. For the TIPPS trial, we were unable to extract baseline characteristics for our group of interest, that is, women with APS. Consequently, the domain deviations from the intended intervention was classified as some concerns. The registered study protocol (ISRCTN87325378) of the FRUIT trial has 1 primary outcome, that is, reduction of preeclampsia before 34 weeks of gestation. The second published primary outcome hypertensive disorder of pregnancy irrespective of gestational age was not prespecified and the selection of the reported result was therefore classified as having some concerns. Considering the limited number of studies included, we did not assess publication bias with a funnel plot.

### 3.4 Included study population

We included 21 women of the subgroup with APS from the TIPPS trial, of which 11 women were randomized to dalteparin and 10 were randomized to the control group. The frequency of aspirin use was not reported for the subgroup of APS women. We included all 32 women from the FRUIT trial, of which 16 were randomized to the dalteparin plus aspirin group and 16 to the aspirin alone group. Baseline characteristics are depicted in Table 2.

**Table 2:** Baseline characteristics.

Study	APS women randomized (*n*)		Gravidity		History of HD		History of SGA		ACA positive according to study definition		Lupus anticoagulant positive		Age (y)		Gestational age (d) at randomization	
	Dalteparin	Control	Dalteparin	Control	Dalteparin	Control	Dalteparin	Control	Dalteparin	Control	Dalteparin	Control	Dalteparin	Control	Dalteparin	Control
TIPPS [19]	11	10	NE	NE	NE	NE	NE	NE	NE	NE	NE	NE	NE	NE	NE	NE
FRUIT [20]	16	16	2.7 ± 1.1	2.6 ± 0.6	11/16 (68.8)	10/16 (62.5)	12/16 (75)	13/16 (81)	14/16 (87.5)	16/16 (81.3)	3/13 (23.1)	4/14 (28.6)	33.6 ± 5.3	30.3 ± 4.2	59.9 ± 15.3	65.9 ± 13.4

Values are *n*/*N* (%) or mean ± SD.

ACA, anticardiolipin antibody; HD, hypertensive disorder of pregnancy; NE, not extractable; SGA, small for gestation age.

### 3.5 Outcomes of this review

The primary outcome of this review, placenta-mediated pregnancy complications, comprising intrauterine growth restriction or birth of an SGA infant, preeclampsia, eclampsia, HELLP syndrome, placental abruption, and late fetal loss, could not be assessed in the TIPPS trial because these data were not reported separately for women with APS. In the FRUIT trial, the primary outcome of this review occurred in 4 of 16 women in the dalteparin group based on the birth of 3 SGA infants and 1 fetal loss after 10 weeks of gestation. Determination of the primary outcome in the control group was hampered by uncertainty on potential coinciding pregnancy complications. In the control group, 3 of 16 women had preeclampsia, HELLP syndrome, or placental abruption, whereas birth of an SGA infant occurred in 3 of 16 women; it is not reported whether the birth of the SGA infants occurred in the same women who had preeclampsia, HELLP syndrome, or placental abruption. Thus, the primary outcome occurred in 3 to 6 of 16 women in the control group (4/16 vs 3/16; RD, 6.25%; 95% CI, −22.3% to 34.8%; ranging to 4/16 vs 6/16; RD, −12.5%; 95% CI, −44.3% to 19.3%).

Components of placenta-mediated pregnancy complications are depicted in Table 3. Secondary outcomes of this review include early pregnancy loss, preterm birth, and maternal adverse events such as bleeding, VTE during pregnancy or puerperium, and heparin-induced thrombocytopenia. Early pregnancy loss and preterm birth were not reported separately for the APS group in the TIPPS trial. In the FRUIT trial, pregnancy loss before 10 weeks of gestation occurred in 1 of 16 women in the dalteparin plus aspirin group compared with 0 of 16 in the aspirin-only group (RD, 6.25%; 95% CI, −5.6% to 18.1%). There were 3 of 16 preterm births in the dalteparin plus aspirin group vs 2 of 16 in the aspirin only group (RD, 6.25%; 95% CI, −18.8% to 31.3%).

**Table 3:** Recurrence of placenta-mediated pregnancy complications.

Study	Pre-eclampsia			HELLP-syndrome			Eclampsia			SGA infant			Placental abruption			Fetal loss > 10 weeks		
	Dalteparin	Control	RD^a^	Dalteparin	Control	RD^a^	Dalteparin	Control	RD^a^	Dalteparin	Control	RD^a^	Dalteparin	Control	RD^a^	Dalteparin	Control	RD^a^
TIPPS [19]	NE	NE	—	NE	NE	—	NE	NE	–	NE	NE	—	NE	NE	—	NE	NE	—
FRUIT [20]	0/16 (0)	2/16 (12.5)	−12.5 (−29 to 3.7)	0/16 (0)	1/16 (6.25)	−6.25 (−18 to 5.6)	0/16 (0)	0/16 (0)	0	3/16 (18.8)	3/16 (18.8)	0 (−27 to 27)	0/16 (0)	1/16 (6.25)	−6.25 (−18 to 5.6)	1/16 (0)	0/16 (0)	6.25 (−5.6 to 18)

HELLP, hemolysis, elevated liver enzymes, and low platelets; NE, not extractable; RD, risk difference; SGA, small for gestational age.

a Values in parenthesis are 95% CI.

In the included APS subgroup of the TIPPS trial, no VTE occurred in the intervention or control group. Major or nonmajor bleeding in women with APS could not be extracted from the TIPPS trial. In the FRUIT trial, no VTE occurred in the intervention or control group. The FRUIT trial did not report major or nonmajor bleeding but did report hematoma without definition in 2 of 16 women in the dalteparin group and none in the control group (RD, 6.25%; 95% CI, −19% to 31%). There were no cases of heparin-induced thrombocytopenia in both trials.

### 3.6 Primary outcome of original studies

The primary study outcome of the TIPPS trial, a composite of VTE, severe or early-onset preeclampsia, birth of an SGA infant, and pregnancy loss occurred in 3 of 11 women in the dalteparin group compared with 3 of 10 women in the control group (RD, 2.7%; 95% CI, −36% to 41%). The primary study outcome of the FRUIT trial, hypertensive disorder of pregnancy with an onset <34 weeks of gestation defined as preeclampsia, eclampsia, or HELLP syndrome occurred in 0 of 16 women in the dalteparin plus aspirin group and in 1 of 16 women in the aspirin-only group (RD, 6.25%; 95% CI, −18% to 5.6%). Hypertensive disorder of pregnancy irrespective of gestational age occurred in 0 of 16 women in the dalteparin plus aspirin group and 2 of 16 women in the aspirin-only group (RD, −12.5%; 95% CI, −28.7% to 3.7%).

## 4 Discussion

In this systematic review, we found insufficient evidence of a beneficial effect of LMWH in women with APS and previous placenta-mediated pregnancy complications. Evidence is limited to only 53 women randomized in 2 RCTs. The TIPPS trial included women with various types of thrombophilia and heterogeneous clinical manifestations, of whom the women with APS was only a small subgroup. The FRUIT trial included only women with APS but was stopped prematurely due to slow recruitment after inclusion of 32 women.

Several guidelines provide weak to moderate recommendations to treat women with APS and a history of placenta-mediated complications with LMWH (Table 4) [[8], [9], [10], [11]]. Our systematic review shows that this is not based on evidence from RCTs, and it appears as if the guidelines have extrapolated evidence from women with APS and recurrent miscarriage, for whom low-quality aggregated evidence from studies with methodological limitations suggests some benefit on the rate of live birth [13] However, recent 2023 ACR/EULAR APS classification criteria underline that recurrent miscarriage as clinical manifestation is not specific enough to be classified as APS. Unlike preeclampsia and placental insufficiency, which are assigned increased weight in APS classification, recurrent miscarriage requires additional clinical manifestations [2].

**Table 4:** Overview of guidelines on treatment of patients with obstetric APS based on late fetal loss or preterm birth due to placental insufficiency.

Guidelines	History of late fetal loss (≥10 wk of gestation)	History of birth <34 wk of gestation due to placental insufficiency
ACCP 2012 [8]	No recommendation	
ACOG 2012 [9]	Prophylactic LMWH + low-dose aspirin should be considered (based on limited or inconsistent scientific evidence, level B)	No recommendation
EULAR 2019 [10]	Heparin at prophylactic dosage in combination with low-dose aspirin is recommended (level 2b recommendation based on level B evidence)	Treatment with low-dose aspirin or low-dose aspirin and heparin at prophylactic dosage is recommended considering the individual’s risk profile (level 2b recommendation based on level B evidence)
ACR 2020 [11]	Strongly recommend combined low-dose aspirin and prophylactic-dose heparin (usually LMWH; strong recommendation based on evidence of moderate strength)	

APS, antiphospholipid syndrome; ACR, American College of Rheumatology; EULAR, European Alliance of Associations for Rheumatology; ACCP, American College of Chest Physicians; ACOG, American College of Obstetricians and Gynecologists; LMWH, low-molecular-weight heparin.

What is the evidence on antithrombotic treatment to prevent (recurrent) placenta-mediated complications in women without APS? Treatment with aspirin reduces the risk the of placenta-mediated pregnancy complications and is recommended in women at high risk, based on a large number of RCTs [21]. For LMWH, however, evidence is limited by smaller RCTs but points into the direction of absence of effect. In an individual-patient data meta-analysis of 8 RCTs in 963 pregnant women with a history of placenta-mediated pregnancy complications defined as preeclampsia, birth of an SGA infant, placental abruption, fetal loss >16 weeks, or recurrent fetal loss >12 weeks, treatment with LMWH did not significantly lower the composite outcome of early onset or severe preeclampsia, birth of an SGA infant, placental abruption, or pregnancy loss ≥20 weeks (14% in LMWH group compared with 22% in the no LMWH group; relative risk, 0.64; *P* = .11) [22]. In addition, in a sensitivity analysis limited to multicenter studies only, the lack of a difference was even more pronounced (18% in LMWH group and 18% in the no LMWH group; RD, −0.6%; 95% CI, −10.4% to 9.2%; *P* = .91). Beyond antithrombotic treatment, immunomodulatory regimens to improve pregnancy outcomes in APS are under evaluation, including a multicenter placebo-controlled RCT that evaluates the effect of hydroxychloroquine in addition to standard treatment [23].

This systematic review has limitations that merit consideration. We were unable to provide a pooled estimate for the prespecified primary outcome because of limited reporting on the subgroup of APS women in the TIPPS trial and uncertainty on coinciding pregnancy complications in the FRUIT trial. With regard to the clinical APS phenotype, inclusion criteria of this review were broader than both the revised Sapporo criteria and the 2023 ACR/EULAR APS classification criteria as we included women with placental abruption and preeclampsia irrespective of gestational age [1,2]. In both the TIPPS and the FRUIT studies, SGA was defined as a birthweight <10th percentile, which is less stringent than the 2023 ACR/EULAR criteria, which define SGA as a birthweight <5th percentile or even < 3rd percentile in absence of other severe features. Moreover, both studies included women with lower antibody titers than defined in the current laboratory APS classification criteria and allowed a <12 weeks or undefined interval between positive tests [2]. The proportion of included women that would have met the 2023 ACR/EULAR criteria is unknown. Discrepancies with the current laboratory APS research criteria appear inherent to trials in APS, as this was also the conclusion from our systematic review on antithrombotic treatment for women with APS and recurrent pregnancy loss [13]. Considering that evidence in women with placenta-mediated pregnancy complications without APLA shows that LMWH is not effective, the mild laboratory phenotype of participants in the TIPPS and FRUIT studies may theoretically have influenced study outcomes toward underestimation of the effect.

This review demonstrates that only 53 women with APS and previous placenta-mediated pregnancy complications have been randomized between LMWH and control and the available evidence is insufficient to support a beneficial effect. Therefore, the present conditional recommendations in guidelines should more clearly underscore the need for future multicenter RCTs, considering the costs, patient burden, and side effects of LMWH injections. The current guidelines hamper the initiation of relevant clinical trials and make it challenging to conduct in this population [24]. However, trials on antithrombotic treatment in pregnancy should and can be done [[25], [26], [27]].

In conclusion, prevention of recurrent placenta-mediated pregnancy complications in APS remains understudied. Clinicians should be aware that data on treatment with LMWH for this indication are insufficient to inform treatment decisions and discuss with women that are prescribed LMWH that this regimen is based on evidence extrapolated from women with APS who had recurrent miscarriage.
