# Supplementary figures and images for: Heparin to prevent recurrent placenta-mediated pregnancy complications in women with antiphospholipid syndrome: a systematic review
Source: Res Pract Thromb Haemost. 2026 May 8;10(4):106634. doi: 10.1016/j.rpth.2026.106634 (PMC13285347; doi:10.1016/j.rpth.2026.106634)

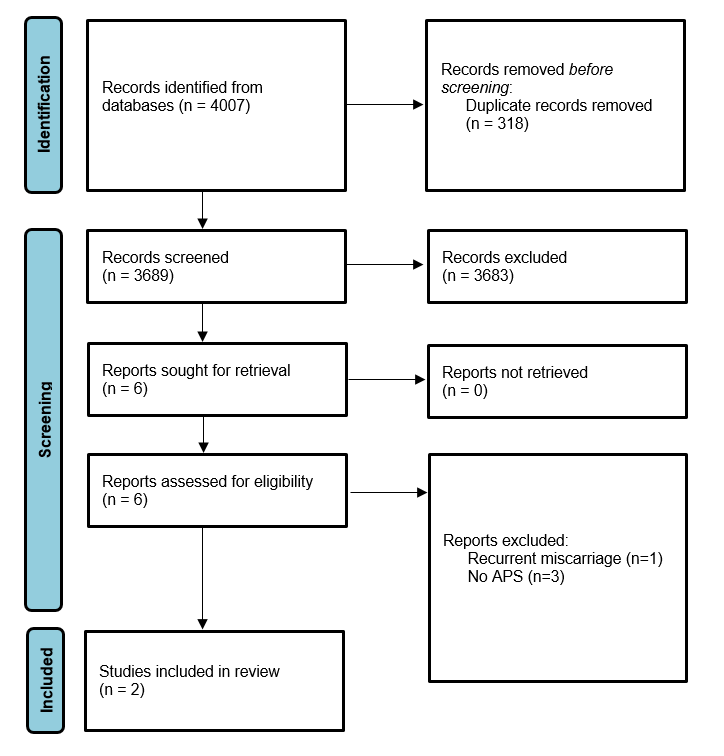
 **Supplementary figure 1: PRISMA 2020 flow diagram of selection of studies**

Supplement: Supplementary Figure 1 [file mmc1.docx]
