# Supplementary material for: Heparin to prevent recurrent placenta-mediated pregnancy complications in women with antiphospholipid syndrome: a systematic review
Source: Res Pract Thromb Haemost. 2026 May 8;10(4):106634. doi: 10.1016/j.rpth.2026.106634 (PMC13285347; doi:10.1016/j.rpth.2026.106634)
Supplement: Supplementary Figure 2 [file mmc2.docx]

|  | Randomization process | Deviations from the intended interventions | Missing outcome data | Measurement of the outcome | Selection of the reported result | Overall |
| --- | --- | --- | --- | --- | --- | --- |
| TIPPS(19) |  |  |  |  |  |  |
| FRUIT (20) |  |  |  |  |  |  |

**Supplementary figure 2: Risk of Bias assessment The Cochrane RoB 2.0**
