# Supplementary material for: Heparin to prevent recurrent placenta-mediated pregnancy complications in women with antiphospholipid syndrome: a systematic review
Source: Res Pract Thromb Haemost. 2026 May 8;10(4):106634. doi: 10.1016/j.rpth.2026.106634 (PMC13285347; doi:10.1016/j.rpth.2026.106634)
Supplement: Supplementary Table 2 [file mmc4.docx]

**Supplementary table S2** Full search strategy in Medline, EMBASE and
The Cochrane Central Register of Controlled Trials.

**Medline**

(Antiphospholipid Syndrome OR Anti-phospholipid syndrome OR Antiphospholipid antibod* OR Anti-phospholipid antibod* OR "Hughes Syndrome" OR "Antibodies, Antiphospholipid"[MeSH] OR "Antiphospholipid Syndrome"[MeSH] OR "Thrombophilia"[Mesh] OR Thrombophil* OR Hypercoagulab*) AND (pregnancy OR gravidity OR parity OR parturition OR obstetric labor OR childbirth OR pre-eclamp* OR preeclamp* OR eclamp* OR hellp OR "placental abruptio" OR "abruptio placentae" OR "solutio placentae" OR "placental insufficiency" OR "intrauterine growth restriction" OR "intrauterine growth retardation" OR "fetal growth restriction" OR "fetal growth retardation" OR "fetal death" OR abortion OR "pregnancy loss" OR "fetal demise" OR iufd OR "Pregnancy"[Mesh] OR "Pregnant People"[Mesh] OR "Pre-Eclampsia"[Mesh] OR "Eclampsia"[Mesh] OR "HELLP Syndrome"[Mesh] OR "Abruptio Placentae"[Mesh] OR "Placental Insufficiency"[Mesh] OR "Infant, Small for Gestational Age"[Mesh] OR "Fetal Growth Retardation"[Mesh] OR "Fetal Death"[Mesh] OR "Abortion, Spontaneous"[Mesh]) AND ("Heparin"[Mesh] OR heparin OR "low molecular weight heparin" OR lmwh OR dalteparin OR tinzaparin OR enoxaparin OR nadroparin OR fragmin OR clexane OR innohep OR fraxiparin) AND (("randomized controlled trial"[Publication Type] OR "controlled clinical trial"[Publication Type] OR "randomized"[Title/Abstract] OR "placebo"[Title/Abstract] OR "drug therapy"[MeSH Subheading] OR "randomly"[Title/Abstract] OR "trial"[Title/Abstract] OR "groups"[Title/Abstract]) NOT ("animals"[MeSH Terms] NOT "humans"[MeSH Terms]))

**Embase**

| **No.** | **Query** |
| --- | --- |
| #61 | #60 AND [embase]/lim NOT ([embase]/lim AND [medline]/lim) |
| #60 | #16 AND #47 AND #59 |
| #59 | #48 OR #49 OR #50 OR #51 OR #52 OR #53 OR #54 OR #55 OR #56 OR #57 OR #58 |
| #58 | fraxiparin |
| #57 | nadroparin |
| #56 | innohep |
| #55 | tinzaparin |
| #54 | clexane |
| #53 | enoxaparin |
| #52 | fragmin |
| #51 | dalteparin |
| #50 | lmwh |
| #49 | low AND molecular AND weight AND heparin |
| #48 | 'heparin'/exp |
| #47 | #17 OR #18 OR #19 OR #20 OR #21 OR #22 OR #23 OR #24 OR #25 OR #26 OR #27 OR #28 OR #29 OR #30 OR #31 OR #32 OR #33 OR #34 OR #35 OR #36 OR #37 OR #38 OR #39 OR #40 OR #41 OR #42 OR #43 OR #44 OR #45 OR #46 |
| #46 | 'iufd' |
| #45 | 'fetal demise' |
| #44 | 'fetal death' |
| #43 | 'pregnancy loss' |
| #42 | 'spontaneous abortion' |
| #41 | 'fetal growth retardation' |
| #40 | 'fetal growth restriction' |
| #39 | 'intrauterine growth retardation' |
| #38 | 'intrauterine growth restriction' |
| #37 | 'placental insufficiency' |
| #36 | 'solutio placentae' |
| #35 | 'placental abruption' |
| #34 | hellp |
| #33 | eclamp* |
| #32 | 'pre-eclamp*' |
| #31 | preeclamp* |
| #30 | childbirth |
| #29 | obstetric AND labor |
| #28 | parturition |
| #27 | parity |
| #26 | gravidity |
| #25 | pregnancy |
| #24 | 'small for gestational age'/exp |
| #23 | 'placenta insufficiency'/exp |
| #22 | 'hellp syndrome'/exp |
| #21 | 'eclampsia and preeclampsia'/exp |
| #20 | 'intrauterine growth retardation'/exp |
| #19 | 'fetus death'/exp |
| #18 | 'abortion'/exp |
| #17 | 'pregnancy'/exp |
| #16 | #1 OR #2 OR #3 OR #4 OR #5 OR #6 OR #7 OR #8 OR #9 OR #10 OR #11 OR #12 OR #13 OR #14 OR #15 |
| #15 | 'hypercoagulability'/exp |
| #14 | hypercoagulab* |
| #13 | thrombophil* |
| #12 | 'thrombophilia'/exp |
| #11 | 'anti phospholipid antibod*' |
| #10 | 'anti-phospholipid antibod*' |
| #9 | 'antiphospholipid antibod*' |
| #8 | 'anti phospholipid syndrome*' |
| #7 | 'anti-phospholipid syndrome*' |
| #6 | 'antiphospholipid syndrome*' |
| #5 | 'anti-phospholipid syndrome*' |
| #4 | 'antiphospholipid syndrome*' |
| #3 | 'hughes syndrome' |
| #2 | 'antiphospholipid syndrome'/exp |
| #1 | 'phospholipid antibody'/exp |

**Cochrane CENTRAL**

| ID | Search |
| --- | --- |
| #1 | Antiphospholipid Syndrome OR "Anti-phospholipid Syndrome" OR Antiphospholipid antibod* OR Anti-phospholipid antibod* OR "Hughes Syndrome" OR Thrombophil* OR Hypercoagulab* |
| #2 | MeSH descriptor: [Antibodies, Antiphospholipid] explode all trees |
| #3 | MeSH descriptor: [Antiphospholipid Syndrome] explode all trees |
| #4 | MeSH descriptor: [Thrombophilia] explode all trees |
| #5 | #1 OR #2 OR #3 OR #4 |
| #6 | MeSH descriptor: [Pregnancy] explode all trees |
| #7 | MeSH descriptor: [Pregnant People] explode all trees |
| #8 | MeSH descriptor: [Pre-Eclampsia] explode all trees |
| #9 | MeSH descriptor: [Eclampsia] explode all trees |
| #10 | MeSH descriptor: [HELLP Syndrome] explode all trees |
| #11 | MeSH descriptor: [Abruptio Placentae] explode all trees |
| #12 | MeSH descriptor: [Placental Insufficiency] explode all trees |
| #13 | MeSH descriptor: [Infant, Small for Gestational Age] explode all trees |
| #14 | MeSH descriptor: [Fetal Growth Retardation] explode all trees |
| #15 | MeSH descriptor: [Fetal Death] explode all trees |
| #16 | MeSH descriptor: [Abortion, Spontaneous] explode all trees |
| #17 | pregnancy OR gravidity OR parity OR parturition OR obstetric labor OR childbirth OR pre-eclamp* OR preeclamp* OR eclamp* OR hellp OR "placental abruptio" OR "abruptio placentae" OR "solutio placentae" OR "placental insufficiency" OR "intrauterine growth restriction" OR "intrauterine growth retardation" OR "fetal growth restriction" OR "fetal growth retardation" OR "fetal death" OR abortion OR "pregnancy loss" OR "fetal demise" OR iufd |
| #18 | #6 OR #7 OR #8 OR #9 OR #10 OR #11 OR #12 OR #13 OR #14 OR #15 OR #16 OR #17 |
| #19 | MeSH descriptor: [Heparin] explode all trees |
| #20 | heparin OR "low molecular weight heparin" OR lmwh OR dalteparin OR tinzaparin OR enoxaparin OR nadroparin OR fragmin OR clexane OR innohep OR fraxiparin |
| #21 | #19 OR #20 |
| #22 | #5 AND #18 AND #21 in Trials |
